# A SCOPING REVIEW OF KNOWLEDGE EVALUATION INSTRUMENTS FOR FORMAL AND INFORMAL DEMENTIA CAREGIVER PROGRAMS

**DOI:** 10.1093/geroni/igz038.2215

**Published:** 2019-11-08

**Authors:** Nicholas V Resciniti, Weizhou Tang, Masroora Tabassum, Dana Al-Hasan, Matthe C Lohman, Mindi Spencer, Daniela Friedman

**Affiliations:** University of South Carolina, Columbia, South Carolina, United States

## Abstract

With the increase in our older adult population there is a need for dementia training for informal and formal dementia caregivers. The objective of this scoping study was to assess dementia knowledge instruments utilized in educational programs and interventions intended for formal and informal dementia caregivers. Scoping review methodology was used to search PubMed, PsycInfo, CINAHL, and Web of Science with tailored database search terms. The search yielded 8,101 results, with 35 studies meeting inclusion criteria. Studies were conducted in eight countries, had varying study designs (RCTs=9, non-RCTs=6, one-group study design=20), and utilized previously published (19) and author-developed (16) instruments. Only two studies focused on minority populations. While author-developed instruments may be more relevant and time-saving, studies should strive to validate instruments or use previously published instruments to help standardize findings across studies and better understand the effects of education programs on caregiver knowledge.

